# Visual Sensory Signals Dominate Tactile Cues during Docked Feeding in Hummingbirds

**DOI:** 10.3389/fnins.2017.00622

**Published:** 2017-11-14

**Authors:** Benjamin Goller, Paolo S. Segre, Kevin M. Middleton, Michael H. Dickinson, Douglas L. Altshuler

**Affiliations:** ^1^Department of Zoology, University of British Columbia, Vancouver, BC, Canada; ^2^Department of Pathology and Anatomical Sciences, University of Missouri, Columbia, MO, United States; ^3^Bioengineering and Biology, California Institute of Technology, Pasadena, CA, United States

**Keywords:** flight control, visual control, sensorimotor integration, tactile perception, Anna's hummingbird

## Abstract

Animals living in and interacting with natural environments must monitor and respond to changing conditions and unpredictable situations. Using information from multiple sensory systems allows them to modify their behavior in response to their dynamic environment but also creates the challenge of integrating different, and potentially contradictory, sources of information for behavior control. Understanding how multiple information streams are integrated to produce flexible and reliable behavior is key to understanding how behavior is controlled in natural settings. Natural settings are rarely still, which challenges animals that require precise body position control, like hummingbirds, which hover while feeding from flowers. Tactile feedback, available only once the hummingbird is docked at the flower, could provide additional information to help maintain its position at the flower. To investigate the role of tactile information for hovering control during feeding, we first asked whether hummingbirds physically interact with a feeder once docked. We quantified physical interactions between docked hummingbirds and a feeder placed in front of a stationary background pattern. Force sensors on the feeder measured a complex time course of loading that reflects the wingbeat frequency and bill movement of feeding hummingbirds, and suggests that they sometimes push against the feeder with their bill. Next, we asked whether the measured tactile interactions were used by feeding hummingbirds to maintain position relative to the feeder. We created two experimental scenarios—one in which the feeder was stationary and the visual background moved and the other where the feeder moved laterally in front of a white background. When the visual background pattern moved, docked hummingbirds pushed significantly harder in the direction of horizontal visual motion. When the feeder moved, and the background was stationary, hummingbirds generated aerodynamic force in the opposite direction of the feeder motion. These results suggest that docked hummingbirds are using visual information about the environment to maintain body position and orientation, and not actively tracking the motion of the feeder. The absence of flower tracking behavior in hummingbirds contrasts with the behavior of hawkmoths, and provides evidence that they rely primarily on the visual background rather than flower-based cues while feeding.

## Introduction

Living in complex and dynamic environments requires animals to integrate—or fuse—information from multiple sensory systems to respond to changing conditions with flexible and effective behaviors. Sensory fusion is a complex process, involving not only dynamic comparisons of different feedback streams, but also constant evaluation of the relevance and reliability of the sensory information in the current behavioral context. Some sensory modalities, like vision, audition, and olfaction, have the capacity to monitor the environment broadly. Others, like touch and the inertial vestibular system, offer more localized or specific information. A key challenge to understanding how a behavior is controlled is to determine what sensory systems inform it and how the information from those sensory systems is integrated.

Animals moving through water (Shaw and Tucker, 1965; Junger and Dahmen, 1991), on land (Lee and Aronson, 1974), and in air (Kelber and Zeil, 1997; Kern and Varjú, 1998; Goller and Altshuler, 2014) have been shown to rely on visual motion—or optic flow—for control of body position (Gibson, 1950). Humans also use tactile and vestibular information for posture control and as perturbations in one of these modalities grow, reliance on the other senses is favored instead (Hwang et al., 2014; Logan et al., 2014). For example, in destabilizing visual conditions, posture stability is improved by finger contact with a nearby stationary object (Holden et al., 1994; Jeka and Lackner, 1994; Jeka et al., 2000; Oie et al., 2002). It is less clear what role tactile information serves for body position control in non-human animals, especially during flight, when there are no ground reaction or buoyancy forces to assist with the estimation of body orientation. Perhaps flying animals decrease their reliance on tactile cues and primarily integrate inertial and visual information to control body motion. Alternatively, tactile information may be a seldom used but highly salient signal for flight control, indicating contact with an external feature and resulting in strong behavioral responses to physical contact.

One flight behavior that involves physical contact with a target is the feeding behavior of nectivorous insects and hummingbirds. Making contact with the flower—or docking—with a proboscis or bill allows the animal to access nectar, but also requires that the hovering visitor maintain proper body position relative to the target. For stationary flowers, the feeding animal only has to dock and maintain a stationary hovering position. However, the behavior is much more complex when the flower moves as on a windy day. In this scenario, tactile feedback from docking could become an important source of information to facilitate the interaction between flower and visiting animal.

Evidence from hawkmoths does not suggest a strong role for tactile information during docked feeding. Hawkmoths dock with a flower using their flexible proboscis, and then use primarily visual information to track flower oscillations up to 5 Hz along the forward-backward axis (Farina et al., 1994) and up to ~3 Hz laterally (Sponberg et al., 2015; Stöckl et al., 2017). Hummingbirds may be similarly reliant on vision, but their rigid bill decreases their positional flexibility while potentially increasing the transmission of physical signals from the flower. Evidence from numerous studies with mammals suggests that different senses are highly interconnected in the vertebrate brain, including integration at early cortical levels (Falchier et al., 2002; Taylor-Clarke et al., 2002; Fu et al., 2003; Rockland and Ojima, 2003). Birds may similarly integrate information at early stages in sensory processing, and certainly do at higher processing levels such as the cerebellum, which is extensively folded as in mammals (Iwaniuk et al., 2006, 2007). Neural and behavioral evidence for extensive sensory integration in vertebrates may translate into behavioral differences between insect and bird flight control mechanisms. What roles do visual and tactile feedback play in controlling the hummingbird's flight position during feeding?

Here, we posed three questions about the role of hummingbird-flower interactions during foraging. We first asked if there is evidence of physical interaction between the flower or feeder and the hummingbird bill. We measured these interactions during docked flight to show that docking could yield a source of tactile information. Next, we asked if hummingbirds use tactile information to override their normal flight response to match the direction of visual background motion. We predicted that rigid tethering to the flower or feeder during docking would make visual and tactile information about the feeder the most reliable stabilization cue, therefore giving it an overriding role in hummingbird position control during docked hovering. Instead, we found that hummingbirds appeared to follow background visual cues as though they were not physically connected to a target feeder. We next asked how well hummingbirds track a feeder moving laterally in front of a blank background. A hummingbird must move laterally to maintain a docked position at a translating feeder and we predicted that visual and tactile signals from the feeder would be used for tracking feeder motion. We measured how wingbeat kinematics differ between stationary feeding and lateral tracking flight, and surprisingly our results suggest that hummingbirds do not track the feeder, unlike hawkmoths. In both stationary and moving feeder scenarios, docked hummingbirds attempted to maintain stationary body position using information from the visual background and not the feeder.

## Experiment 1

### Motivation

We investigated the physical interactions between the hummingbird bill and the target food source by measuring forces exerted on the feeder by the docked hummingbird in front of stationary grating or dot-field patterns. The force measurements revealed several signatures of docking, so we tested the prediction that the tactile signals from docking at the feeder would override hummingbird flight responses to visual motion (Goller and Altshuler, 2014). Forces exerted on the stationary feeder by the docked hummingbird were again measured, but this time we moved the background grating or dot-field patterns during feeding. Counter to our prediction, hummingbirds responded to visual motion by pushing against the feeder in the direction of the moving background pattern.

## Materials and methods

### Animals

We captured eight male Anna's hummingbirds (*Calypte anna*) on the campus of the University of British Columbia using drop-door traps (Russell and Russell, 2001). All hummingbirds were housed in individual cages and fed *ad libitum* on solutions of artificial nectar (Nektar-Plus, Nekton, Pforzheim, Germany) and sugar. Before the experiments, the birds were allowed to acclimate to the flight chambers. The procedures for Experiment 1 were approved by the Animal Care Committee at the University of British Columbia.

### Feeder force sensor rig and training

**Figure 1:**
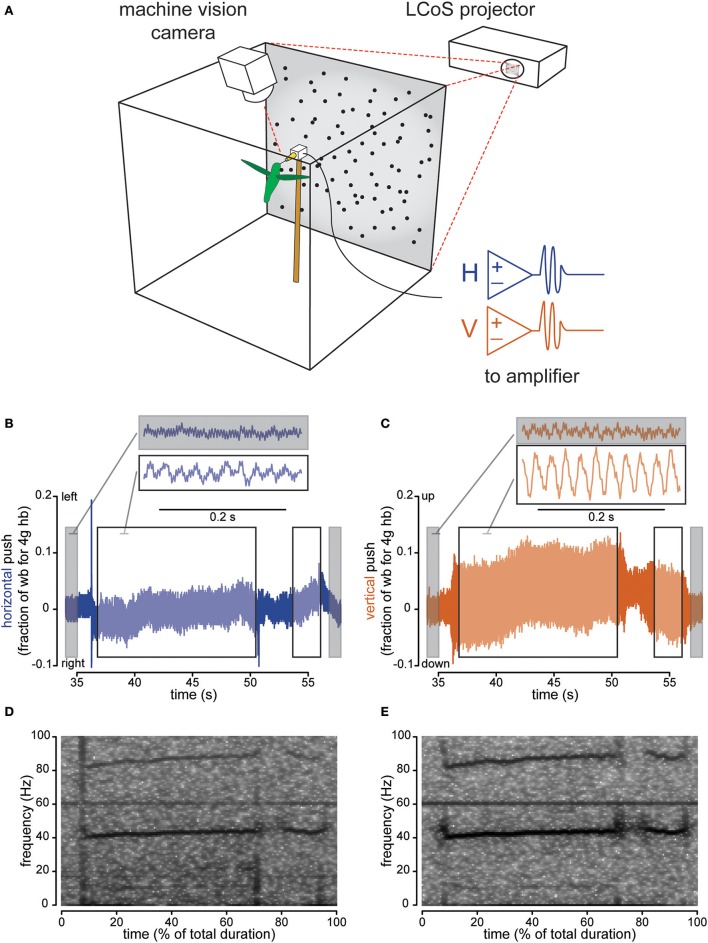
Instrumented feeder measures how hovering hummingbirds interact with the feeder once docked. The flight chamber **(A)** is a 0.5 m cube with a back-projection screen on one wall and a feeder centered in front of it. Horizontal and vertical strain, as well as video, are recorded and computer generated visual stimulus is presented behind the feeder. Strain recordings show pushing against the feeder in body weight units (assuming an average 4 g Anna's hummingbird) for both left-right (horizontal axis, **B**) and up-down (vertical axis, **C**) pushes. Gray highlights sections when the bird is not present, while the black-bordered white box indicates sections when the bird is docked. The two traces shown are examples where the background is stationary. Spectral analysis of the horizontal **(D)** and vertical **(E)** axis traces (**B,C** respectively) show the appearance of the wingbeat (~40 Hz) and more faint licking (10–15 Hz) frequency bands when the bird is docked. For Figures 1–**3**, the horizontal axis is colored with blue and the vertical axis is colored copper-red.

The experiment chamber consisted of an acrylic cube with 0.5 × 0.5 m wall panels (Figure 1A). One side panel was coated with a frosted window coating (wallpaperforwindows.com) to allow back-projection with a Canon REALiS SX80 Mark II projector (3,000-lumen lamp, 1,400 × 1,050 SXGA, 60 Hz). A feeder was centered 23 cm in front of the projection screen and filled with 0.6 ml of 15% by weight table sugar solution. The open floor of the cube was covered with nylon mesh to provide a path for wing downwash. The insides of the three walls without projection were covered with black paper to prevent reflections of visual stimuli. Behavior was monitored at 10 fps using an AVT GigE680 machine vision camera (Allied Vision, Exton, PA) above the clear ceiling and the video stream was recorded using Streampix 7 software (NorPix, Inc., Montreal, Canada). The cube face to the feeding bird's right contained a door to enable access to the feeder between trials.

The feeder was a 0.6 ml sugar reservoir mounted on a custom-built sensor that measured strain in both the horizontal and vertical axes. The sensor was PLA plastic components printed using an Ultimaker2 (Ultimaker B.V., Geldermalsen, Netherlands), and connected by four 13 × 5 mm pieces of 0.003″ 18-8 stainless steel shim stock. Each shim stock piece was mounted with a N2A-13-S071P-350/LE2 model strain gauge using MBond 610 adhesive (Vishay Precision Group, Malvern, PA). Two shim stock-gauge pieces were mounted so that they transduced lateral deflections; two were mounted so that they transduced vertical deflections. The paired gauges in each direction were wired on opposing arms of a Wheatstone bridge, with the other arms completed with 350 Ω resistors. The horizontal and vertical bridges were powered by separate 9V batteries and voltage signals were amplified 5,000x and 20,000x respectively with a Brownlee Precision Model 440 four-channel amplifier (NeuroPhase LLC, Santa Clara, CA) to achieve similar baseline signal amplitudes. Amplified voltage signals were recorded at 1,000 Hz using a NI USB-6009 board (National Instruments Corp., Austin, TX) and custom written scripts in Matlab R2013b using the Data Acquisition Toolbox (The MathWorks Inc., Natick, MA). An event channel was also recorded to synchronize the strain recordings with stimulus onset.

Two types of patterns were used to create visual motion on the 0.5 × 0.5 m projection surface at a resolution of 1,050 by 1,050 pixels. The docked hummingbird's head was 0.25 m from the background surface, which covered approximately 90° of the frontal visual field, horizontally and vertically. One background pattern was a square-wave, black-and-white grating with four sets of either vertical or horizontal bars (spatial frequency: 0.044 cycles/°), similar to the low spatial frequency gratings used in a previous study (Goller and Altshuler, 2014). The other pattern was a random dot-field with 250 black dots (40 px or 3.4° diameter) on a white background, and was similar to patterns used in electrophysiological studies of motion perception (Gaede et al., 2017). Dots were randomly positioned, had infinite lifetimes, and would regenerate at the origin of motion once they moved off-screen. Stimuli were generated and controlled using custom Matlab scripts using Psychtoolbox-3 (Brainard, 1997; Pelli, 1997; Kleiner et al., 2007).

Hummingbirds were trained in the experimental chamber with a non-moving pattern projection. Four birds were trained with a grating and four with a dot-field. Birds were trained to feed on a 15-min schedule by blocking access to sugar for 15 min, then opening the feeder for up to 2 min. No birds were excluded from experiments on the basis of failed training. After training, two experiments were conducted on two separate days within a 4-day span—one varying the direction of visual motion, the other varying the motion speed. The first experiment was to move the background pattern in four different directions (left, right, up, and down) at 12°/s. Birds were tested with their training pattern. Each direction was tested twice, along with two non-moving trials, in random order. At the end of each trial, the feeder was refilled from a syringe to measure feeding volume. After the initial set of 10 stimulus treatments, trials where subjects fed less than 0.1 ml were repeated.

In the second experiment, we varied the speed of visual motion to investigate possible speed tuning of responses to visual motion during docked hovering. We used the same procedure as the directional experiment but with different stimuli. In the speed experiment, all birds were tested with both patterns. The dot-field or grating pattern was moved to the left at seven different speeds: 0, 2, 12, 48, 72, 84, 120°/s. The range of speeds from 0 to 120°/s spans the range tested in an electrophysiological study with Anna's hummingbirds, zebra finches, and pigeons, and which showed that hummingbird neurons were tuned for the faster speeds tested (Gaede et al., 2017). Each speed and pattern combination was used once. Again, trials where the bird did not drink at least 0.1 ml were repeated after the predetermined random trial sequence had been completed.

### Force measurement statistical analysis

Bird presence at the feeder was determined by digitizing video recordings. The timestamps of the bird arriving and leaving were matched to the recorded traces from the horizontal and vertical strain amplifier channels (Figures 1B,C). Portions where the bird was present and feeding were conservatively cropped by 0.25 s on either end to eliminate signal spikes caused by docking and undocking with the feeder. The feeding portions were analyzed relative to a minimum of 3 s of recording before the first feed and after the last. Each trace was detrended by subtracting a linear fit to the pre- and post-feed data. Strain signals were calibrated using weights of 2, 5, and 10 g, with the feeder reoriented appropriately. Calibration points were analyzed and fit with a line through the origin. We normalized the force measurements to the average body weight (4.0 g) of local hummingbirds. Trace analysis and statistical analysis was performed in R version 3.1.3 (R Core Team, 2015) using linear mixed models (Pinheiro et al., 2016) and *post-hoc* testing using Tukey tests in the multcomp package (Hothorn et al., 2008). Fixed effects included pattern, stimulus speed, and stimulus motion direction. Individual hummingbirds, and repeats of trials within individual were treated as random effects. Spectral analysis was performed with the signal package (Signal Developers, 2013).

## Results

### Hummingbirds physically interact with the feeder during feeding

**Figure 2:**
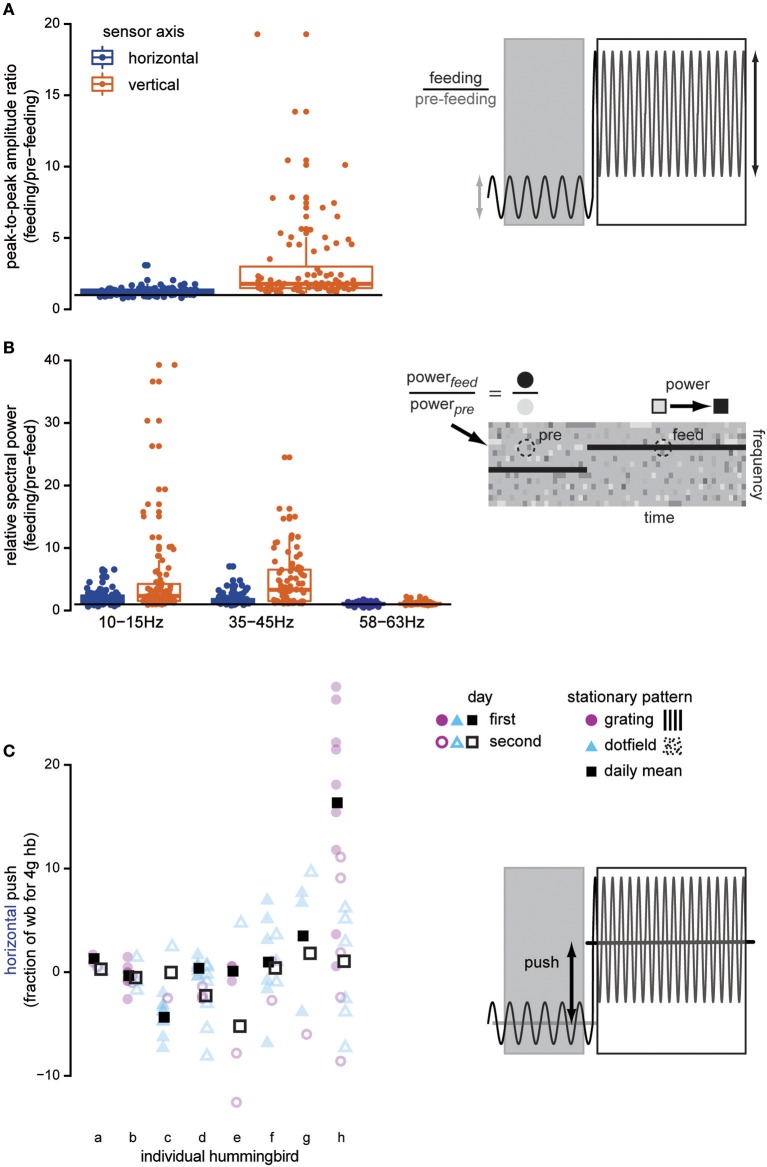
Docked hummingbirds interact with the feeder. Data shown here are from trials where stationary dot-fields and gratings are presented. Once the bird docks, both the amplitude and the frequency of oscillations in the force signals increase above background noise levels, indicating that the feeding hummingbird is exerting forces on the feeder. The peak-to-peak amplitude of the recording **(A)** increases when the hummingbird docks, especially in the vertical axis (copper). The horizontal line illustrates a ratio equal to 1, indicating no change in the signal after the bird has docked. We compared the spectral power of various frequencies in the feeding and pre-feeding strain measurements **(B)** to determine if behaviorally relevant frequencies changed once the hummingbird docked (visually illustrated by comparing the colors of the dotted circle regions). Our measurements exhibit signatures of two behaviorally relevant frequencies: licking frequency (10–15 Hz) and wingbeat frequency (35–45 Hz). In comparison to the electrical noise that is not bird-related, these frequencies appear in the strain recordings after the bird docks and suggest that the bird's bill transmits body motions. Finally, the bird also adjusts its body or head position to push against the feeder in the horizontal (blue) axis **(C)**. This type of interaction with the feeder shifts the overall mean of the force signal, in contrast with the previous measures which cause changes in the signal oscillation. Pushing is highly variable both within and between individuals, but there is no systematic trend across multiple experiment days (filled vs. open) or between background visual patterns (purple vs. blue). The faded symbols show the raw data, and the full-color squares show daily means.

Hummingbirds physically touch the feeder when they are docked, suggesting that tactile feedback could play a role in hovering control. We measured forces exerted on the feeder using a sensor with separate horizontal and vertical channels. Before the bird docked, the signal recorded at the feeder was characterized by low-amplitude oscillations, primarily 60 Hz electrical noise (Figures 1B,C for representative raw traces, Figures 1D,E for spectrograms). After docking, the signal increased in amplitude and the frequency of the oscillations changed, indicating that the feeding hummingbirds were transmitting forces to the feeder. For each trial with a stationary background, we calculated the ratio of the peak-to-peak signal amplitude before the bird docked to the value after the bird docked (Figures 1B,C for traces, Figure 2A for analysis). The amplitude ratios for the vertical axis always increased (min = 1.141, median = 1.783) demonstrating that hummingbirds touch the feeder once docked. We recorded less change in the horizontal axis (min = 0.775, h median = 1.211), which exhibits a significantly lower amplitude ratio than the vertical axis [*F*_(1, 100)_ = 44.428, *P* < 0.0001] by about 2.4 times (amplitude ratio means: vertical = 3.000; horizontal = 1.236).

The changes in the force measurements during docked feeding are related to several behaviorally relevant frequencies (Figures 1D,E for representative spectrograms, Figure 2B for analysis). Anna's hummingbirds open their bill tips and extend their tongue to lick nectar at about 10–15 Hz (Ewald and Williams, 1982; Rico-Guevara and Rubega, 2011) and we used spectral analyses to determine whether the licking frequency was measured at the feeder. We calculated the spectral power of the force signal in the 10–15 Hz range and compared the power before the bird arrived to after it docked. After docking, the power of the signal in the licking frequency range increased (ratio means: vertical = 4.991; horizontal = 1.956). Hovering wingbeat frequency around 35–45 Hz (Clark and Dudley, 2010; Altshuler et al., 2012) is also transmitted to the feeder, again shown by an increase in 35–45 Hz spectral power ratio between the signal before the hummingbird arrives and after it docks (ratio means: vertical = 4.683; horizontal = 1.768). For comparison, the power of the signal at 60 Hz, electrical background noise, is relatively unchanged (ratio means: vertical = 1.096; horizontal = 1.047). The licking and wingbeat frequencies are transmitted significantly more in the vertical axis than the horizontal axis [10–15 Hz: *F*_(1, 100)_ = 17.725, *P* < 0.0001; 35–45 Hz: *F*_(1, 100)_ = 75.862, *P* < 0.0001] but not the electrical noise [60 Hz: *F*_(1, 100)_ = 2.616, *P* = 0.109].

In addition to the high frequency loading, hummingbirds also push against the feeder when the background is stationary. Pushing is measured as the difference between the signal mean during feeding and the pre-feeding signal mean. Individual hummingbirds vary in how much they push against the feeder during feeding and how much variance they exhibit from feed to feed (Figure 2C). However, this is not systematic and there is no effect of pattern [*F*_(1, 52)_ = 1.432, *P* = 0.237] or day [*F*_(1, 19)_ = 0.791, *P* = 0.385] on the magnitude of this horizontal load. Similarly, the vertical load is not affected by stationary background pattern [*F*_(1, 52)_ = 0.788, *P* = 0.389] or day [*F*_(1, 19)_ = 1.982, *P* = 0.175].

### Hummingbirds stabilize directional visual motion during feeding

**Figure 3:**
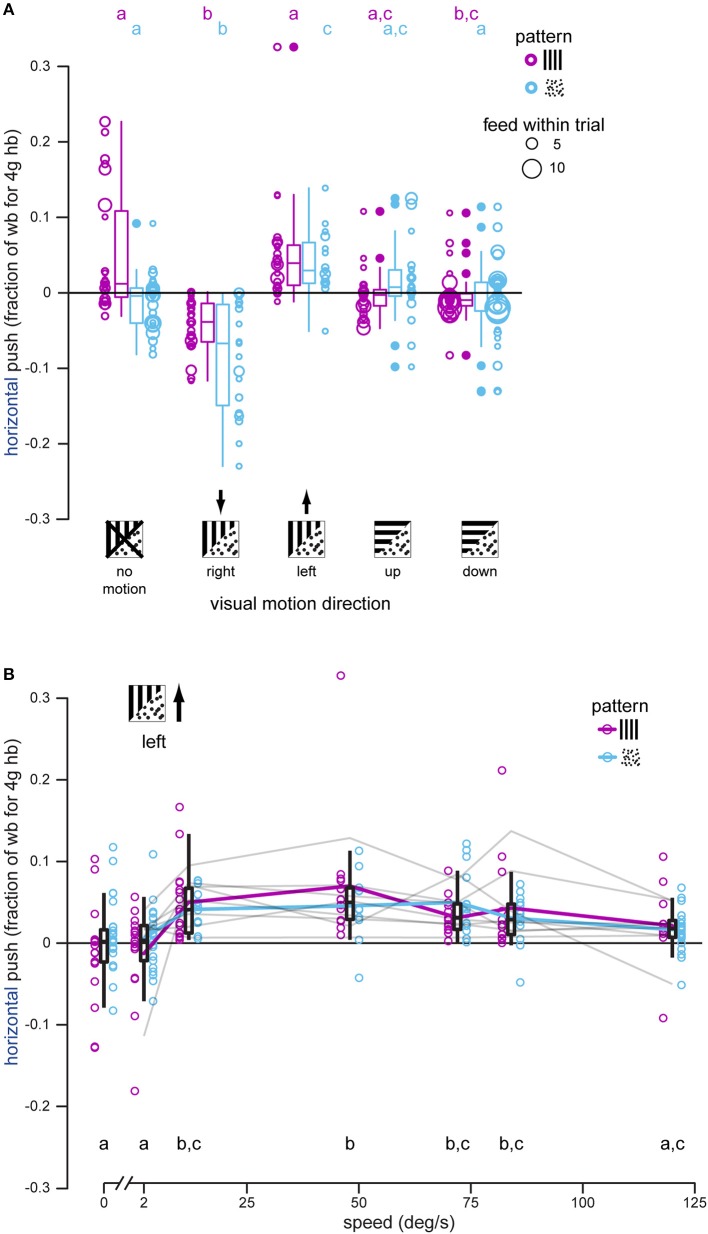
Hummingbirds attempt to stabilize lateral visual motion during docked feeding, especially at intermediate motion speeds. **(A)** Horizontal push was measured each time a hummingbird docked, and each bird was presented with right-, left-, up-, and down-moving patterns, as well as stationary ones, during feeding. Black arrows indicate the expected push direction if the bird attempts to stabilize the directional visual motion. Gratings had significantly less of an effect than dot-fields. In most cases hummingbirds docked multiple times within a single stimulus trial (multiple feeds within a trial) and this is indicated by the increasing diameter of the raw data symbols. Summary statistics of all feeds in a stimulus and pattern group are shown by box-and-whisker plots. Hummingbirds push against the feeder to stabilize right and left dot-field motion, and only significantly push to the right for grating motion. There is no consistent horizontal push for vertical motions. **(B)** Horizontal push was measured for docked hummingbirds feeding in front of gratings and dot-fields moving to the bird's left at different speeds from 0 to 120°/s. Raw data are shown along with summary box-and-whisker plots. Although responses to the two patterns are not statistically different, pattern means are illustrated by colored lines to visualize the tuning curve. The individual tuning curves for each of the eight individual birds are shown in gray (combined responses for the two patterns). Overall, birds exhibit a response plateau by 12°/s that then declines for motion faster than 84°/s. No response was measured for patterns moving at 2°/s.

Hummingbirds feeding in front of a moving background push against a stationary feeder in the direction of visual motion but remain docked. We analyzed horizontal load (Figure 3A) using a linear mixed effects model and found an interaction between pattern and stimulus motion direction [*F*_(4, 125)_ = 3.279, *P* = 0.0136]. Splitting the analysis by pattern shows that stimulus motion direction has an effect on the measured horizontal load for both gratings [*F*_(4, 68)_ = 13.120, *P* < 0.0001] and dot-fields [*F*_(4, 57)_ = 14.305, *P* < 0.0001]. *Post-hoc* Tukey tests were used to compare the load magnitudes (in fractions of body weight for a 4 g hummingbird) and we report the comparisons with stationary backgrounds. *Post-hoc* comparisons are reported as a mean response, a difference in means between motion treatment and stationary treatment as an effect size, and significance. A positive sign indicates either left or up and a negative sign indicates right or down. For example, during stationary dot-field trials birds pushed slightly right on average (−0.0065 wb), whereas they pushed more strongly to the left for non-moving grating trials (0.033 wb).

Dot-fields moving horizontally elicited horizontal loading in docked hummingbirds (right: −0.0818, −0.0753 wb, *P* < 0.001; left: 0.0397, 0.0462 wb, *P* = 0.015). Gratings also elicited loading to the right (−0.0419, −0.0750 wb, *P* < 0.001) and left (0.0476, 0.0145 wb, *P* = 0.981) although the leftward response is not significant in comparison with that measured during the no-motion trials. Horizontal load during vertical motion trials was close to zero for both gratings (up: 0.00143, −0.0316 wb, *P* = 0.111; down: −0.00300, −0.0360 wb, *P* = 0.00495) and dot-fields (up: 0.00397, 0.0105 wb, *P* = 0.442; down: −0.0137, −0.00718 wb, *P* = 0.998), suggesting that lateral loading of the feeder is a response to lateral visual motion, and not visual motion in general.

We do not present the vertical axis data here because of issues with our daily calibration of the signal from the vertical axis strain gauges. Calibration slopes relating signal voltage to mass for the vertical axis were bimodally distributed with groups around 0.0487 and 0.433 V/g, for an overall mean of 0.164 ± 0.192 V/g (±s.d.). In contrast, the horizontal axis had a consistent set of calibration slopes throughout the experiment for a mean of 0.159 ± 0.0454 V/g. We found that the apparent fluctuation in the vertical channel happened unpredictably during some trials, but the peak-to-peak amplitude of pre-docked force measurements does not exhibit a clear bimodal distribution like the calibration slopes. We therefore do not have the information to retroactively determine which calibration values to use for which recordings. Perhaps the signal amplifier may have failed to maintain consistent signal amplification for the vertical measurements. We therefore decided to omit the vertical push data from the pattern direction and speed analyses.

### Visual motion stabilizing response in feeding hummingbirds is speed tuned

We also tested a single motion direction at a range of speeds to determine if the lateral loading of the feeder, caused by the hummingbird in response to lateral visual motion, varied with speed. Speed dependence was again analyzed with a linear mixed effects model, this time using speed as the fixed effect instead of direction. All visual motion was to the left in this experiment, so a leftward (positive) load was predicted. Analysis of the horizontal load yielded a significant effect of speed [*F*_(6, 166)_ = 10.118, *P* < 0.0001] but not pattern [*F*_(1, 166)_ = 2.516, *P* = 0.115] and no interaction between speed and pattern [*F*_(6, 166)_ = 1.276, *P* = 0.271]. We removed the interaction term to allow a *post-hoc* Tukey test to determine differences between speeds (Figure 3B). For trials with a stationary pattern, hummingbirds pushed very little against the feeder (mean = 0.000392 wb). Trials with leftward motion at speeds of 2 and 120°/s were not significantly different from no motion (2°/s: −0.00169, −0.00209 wb, *P* = 1.0; 120°/s: 0.0128, 0.0124 wb, *P* = 0.247). Leftward motion at speeds of 12–84°/s elicited significant pushing to the left (push range 0.0453–0.0503 wb, difference range 0.0449–0.0499 wb, *P* < 0.00475). Over the range tested, the birds' responses were maximal between 12 and 48°/s. For comparison, the feeder used in the next section was moved laterally at 0.15 m/s, and a docked hummingbird matching the lateral translation of the feeder would experience horizontal visual motion of approximately 30°/s.

## Experiment 2

### Motivation

Hummingbirds docked at a stationary feeder attempted to move in the direction of visual background motion, suggesting that they prioritize sensory information from the background, over that from the flower. We next asked how hummingbirds might maintain docked position at a wind-blown flower or moving feeder, without a moving visual background. Again, we predicted that hummingbirds would follow sensory cues from the feeder, much like a hawkmoth, instead of cues from the background. We could not move the feeder equipped with strain gauges (Experiment 1) without making the force measurements unusable because of the resonant oscillations. Therefore, in lieu of direct force measurements, we described wingbeat kinematics for hummingbirds feeding from stationary and laterally translating feeders and used quasi-steady modeling to determine the direction and magnitude of forces produced by the kinematics. We again found that hummingbirds did not fly to precisely match their position to the position of the feeder, but instead produced forces opposing the motion of the feeder.

## Materials and methods

### Animals

We captured two male Anna's hummingbirds (*Calypte anna*) on the campus of the University of British Columbia and six adult male Anna's hummingbirds near the campus of the California Institute of Technology using drop-door traps (Russell and Russell, 2001). The birds captured in British Columbia were used for flow visualization. The California-caught hummingbirds were used for kinematic experiments and because they were larger than the birds in British Columbia, we report force magnitudes as a fraction of body weight using the greater body mass of 4.68 g in this experiment. All details for housing and feeding the hummingbirds as in Experiment 1. The procedures for Experiment 2 were approved by the Animal Care Committee at the University of British Columbia and the Institutional Animal Care and Use Committee of the California Institute of Technology.

### Moving feeder rig and training

**Figure 4:**
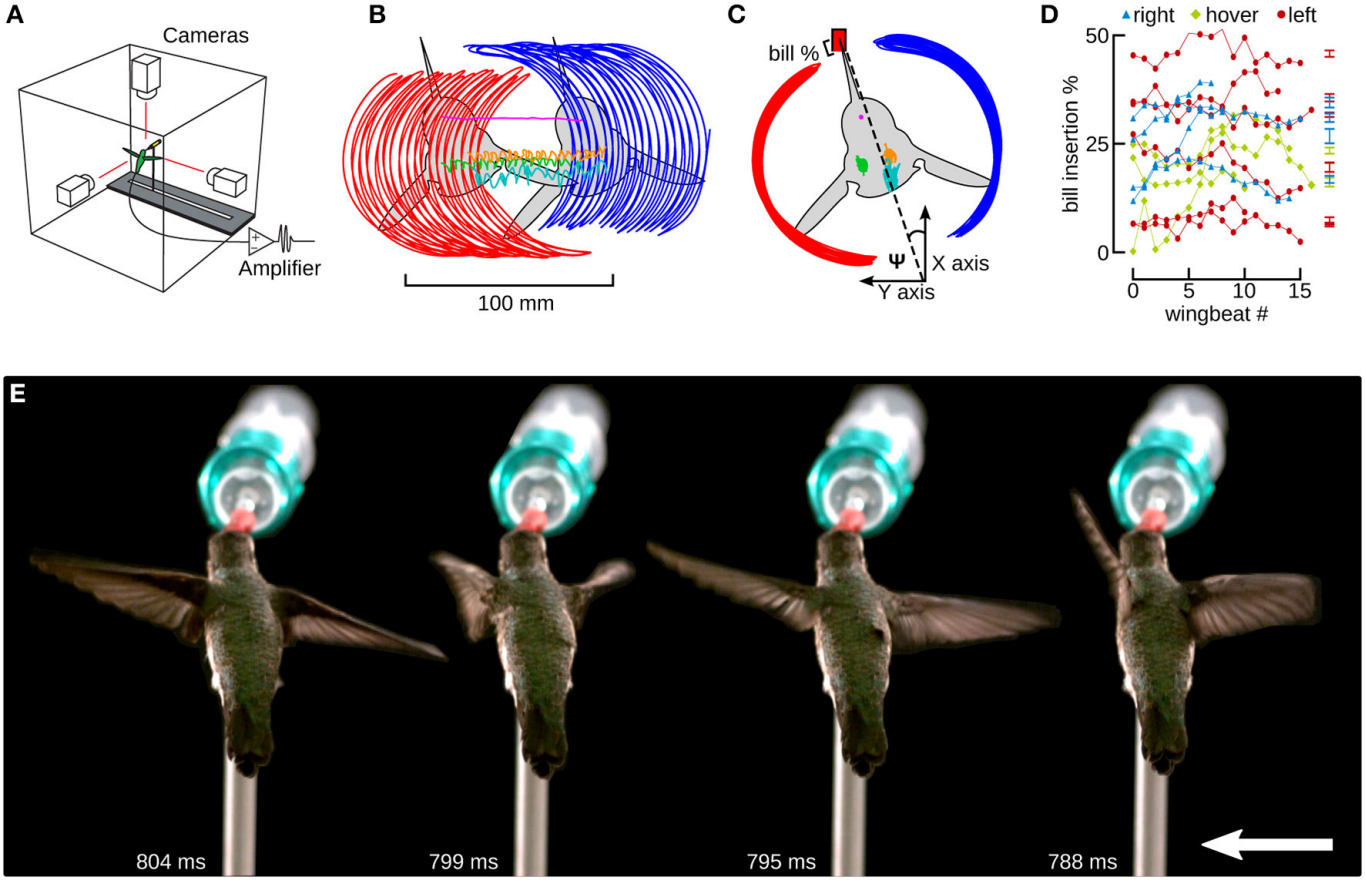
Hummingbirds track a feeder moving laterally by inserting their bill by a similar amount as during hovering flight. **(A)** Hummingbird feeder tracking was studied in an acrylic flight chamber. The feeder was moved left and right using a stepper motor and linear slide. Three high-speed cameras were placed orthogonally and filmed the bird during hovering and feeder tracking. Trailing EMG electrodes were connected to an extracellular amplifier just outside of the chamber. **(B)** A top view of a bird moving laterally to the left. Six digitized anatomical features are presented in this panel: left wingtip (red), right wingtip (blue), left shoulder (green), right shoulder (orange), head (magenta), and tail (cyan). The colors red and blue indicate left and right in Figures 4–**6**. **(C)** The frame of reference was transformed by aligning every frame to the head, and every wing stroke to the midpoints between the stroke transitions. The travel angle Ψ is the angle between the wingtip path dividing line and the orientation of the feeder, which is aligned with the x-axis. Ψ is positive when the bird is facing the direction of motion and negative when it is facing away. The bill % is the percentage of the exposed culmen inserted into the feeder. **(D)** The bill insertion percentages are plotted for all hovering (green), and left (red) and right (blue) lateral flight trials. **(E)** A time lapse of images from one wingbeat during feeder tracking to the left are spread out for clarity. The downstroke begins at 788 ms (white text) and the mid-downstroke occurs at 795 ms. The upstroke begins at 799 ms and the mid-upstroke occurs at 804 ms. The distance between each pair of images is equivalent to the distance the bird and feeder would move over 20 wingbeats. The feeder was moving at 15 cm/s. The entire video from which this sequence is obtained is available in the online Supplementary Materials (Video S1).

Feeder tracking experiments were performed in an acrylic cube (0.9 m^3^) with three clear sides and three white sides to provide backgrounds for filming (Figure 4A). The birds were trained to feed from an artificial feeder made out of a 25 ml syringe mounted on a 45 cm linear slide table powered by a stepper motor (X18 and MD2b, Arrick Robotics, Tyler TX). The opening of the feeder was the same diameter as the opening of the instrumented feeder in Experiment 1. The feeder was oriented horizontally and 90° to the axis of table motion so that the hummingbirds needed to perform lateral flight during feeder movement. The birds were trained to feed on command once every 20 min (Altshuler et al., 2012). Six birds were filmed during hovering and while moving laterally at 0.15 m/s in both the left and right directions. This lateral motion speed was chosen to maximize the need for horizontal force production, and although some birds were able to track at faster speeds, we selected the speed where all individuals were able to match the motion of the feeder. These trials included electromyography (EMG) measurements, but we do not present analysis of the muscle recordings here. The electrode wires loosely trailed the flying hummingbird, so we consider the flight in Experiment 2 as untethered flight.

The trials were recorded using three synchronized high-speed cameras (Photron APX, San Diego CA; fps: 1,000 frames/s, shutter: 1/6,000 s). The cameras were placed posterior, lateral, and dorsal relative to the bird's anticipated location during feeding. Calibration was performed using direct linear transformation (DLT) with a 27-point calibration object in DLTdv5 software (Hedrick, 2008). Eight points were digitized on each hummingbird: right shoulder, left shoulder, right wing tip, left wing tip, right 5th primary, left 5th primary, top of the head, and the tip of the middle tail feather. The 2D points were transformed to 3D coordinates that were filtered with a zero-phase, fourth-order low-pass Butterworth filter. The filter cut-off frequencies were six times the wingbeat frequency for the shoulders and tail, eight times the wingbeat frequency for the wingtip and 5th primary, and twice the wingbeat frequency for the head. We defined the pronation time for each wing as the time of minimum excursion in the stroke plane and the supination time as the time of the maximum excursion.

To supplement and confirm the kinematic analysis, we recorded two separate hummingbirds in flow visualization trials to determine the orientation of wing-generated forces. The flow visualization trials were performed with using a different stepper motor and linear actuator (M-drive 23 Motion Control, Schneider Electric, Marlborough CT). The birds were filmed performing controlled lateral flight at 15 and 30 cm/s while flying through a plume of CO_2_ created by dropping dry ice cubes into hot water. This method is described in detail in Pournazeri et al. (2013). The trials were filmed from the posterior perspective using a single camera (M120, Vision Research, Wayne NJ, USA; fps: 1,000 frames/s, shutter: 1/1,110 s).

### Body and wingbeat kinematics

To compare the kinematics across wingbeats, we used a gravitational frame of reference defined by the position of the wings at the start and end of downstroke and upstroke (Figures 4B,C). The frame of reference and the kinematic variables are described in detail elsewhere (Altshuler et al., 2012; Read et al., 2016). Briefly, we focus in this study on two variables to describe body angle (lateral body angle [χ_GR, XZ]_, frontal body angle [χ_GR, YZ_]), and two variables to describe the position of the wing stroke (wing bank angle [*WBA*], relative wing bank angle [*RWBA*]). *WBA* is defined as the difference in the average elevation angle between the left and right wings, divided by 2. By convention, when the left wing is elevated and the right wing is depressed the *WBA* is positive. *RWBA* is a measure of the wing bank relative to the frontal body axis and is calculated as the absolute value of the sum of the *WBA* and χ_GR, YZ_. When the wings are perpendicular to the body axis the *RWBA* is zero. We also calculated the geometric angle of attack (α) as the angle between the plane of the wing—defined by three points: shoulder, wingtip, and tip of the 5th primary—and the horizontal for a given wing elevation. By this convention, a geometric angle of 0° signifies that the wing is oriented parallel to horizontal, and at 90° the wing is oriented vertically. The bill insertion (Bill %) is the percentage of the exposed culmen inside the artificial feeder, calculated using ImageJ software. The frame of reference transformations and the calculations of the kinematic variables were made using custom software written in Python (Python Software Foundation, Wilmington, DE).

To quantify the changes in kinematics we used a mixed-model ANOVA with direction of travel (right lateral, hover, left lateral) as the fixed effect and bird as random effect, and when the results were significant (α = 0.05) we performed the three *post-hoc* comparisons to test for significant differences between right lateral flight and hovering, left lateral flight and hovering, and right and left lateral flight.

### Quasi-steady aerodynamic force analysis

To quantify the aerodynamic effects of the wingbeat kinematics for each treatment, we used a quasi-steady state aerodynamic analysis based on the blade-element model developed by Weis-Fogh (1973) and employed by others (e.g., Fry et al., 2005; Kruyt et al., 2014). This approach integrates the instantaneous lift and drag forces occurring along chord-wise sections (blades) of the wing over the time course of the wingstroke. By definition, quasi-steady state aerodynamic models do not include rotational lift generating mechanisms that are known to act on reciprocating wings (Dickinson et al., 1999; Altshuler et al., 2005). The instantaneous, quasi-steady lift and drag acting on each flapping wing are calculated as:

(1) $$\vec{Lift}=1{/}_{2}\;C_{\text{L}}\;\rho \;S{\vec{V}}_{\text{incident}}^{2}$$

(2) $$\vec{Drag}=1{/}_{2}\;C_{\text{D}}\;\rho \;S{\vec{V}}_{\text{incident}}^{2}$$

where ρ is the air density (1.18 kg m^−3^) and *S* is the surface area of the wing (6.78E^−4^ m^2^). The wing and body mass measurements used in the model are species-averaged values for *C. anna* that were reported in a previous study (Kruyt et al., 2014). *C*_L_ and *C*_D_ are the coefficients of lift and drag, respectively, which were calculated using the aerodynamic angle of attack (α_aero_, °), defined as the angle of attack of the wing relative to the incident velocity (*V*_incident_):

(3) $$C_{\text{L},\text{ down}}=0.0031\;+\;1.5842\;\times \text{ cos}(0.0301\alpha_{\text{aero}}\;+\;4.7124)\;$$

(4) $$C_{\text{D},\text{ down}}=8.3171\;+\;8.1909\;\times \text{ cos}(0.0073\alpha_{\text{aero}}\;+\;3.1416)\;$$

(5) $$\begin{array}{l} C_{\text{L},\text{ up}}=0.0028\;+\;1.1251\;\times \text{ cos}(0.0332(\alpha_{\text{aero}}-180)\\ \;+\;4.6963) \end{array}$$

(6) $$\begin{array}{l} C_{\text{D},\text{ up}}=1.1993\;+\;1.0938\;\times \text{ cos}(0.0281(\alpha_{\text{aero}}-180)\\ \;+\;3.1277) \end{array}$$

These equations were empirically derived for *C. anna* wings and presented in Kruyt et al. (2014), and differ here only in the sign convention of α_aero_ with respect to the up- and down- strokes.

**Figure 5:**
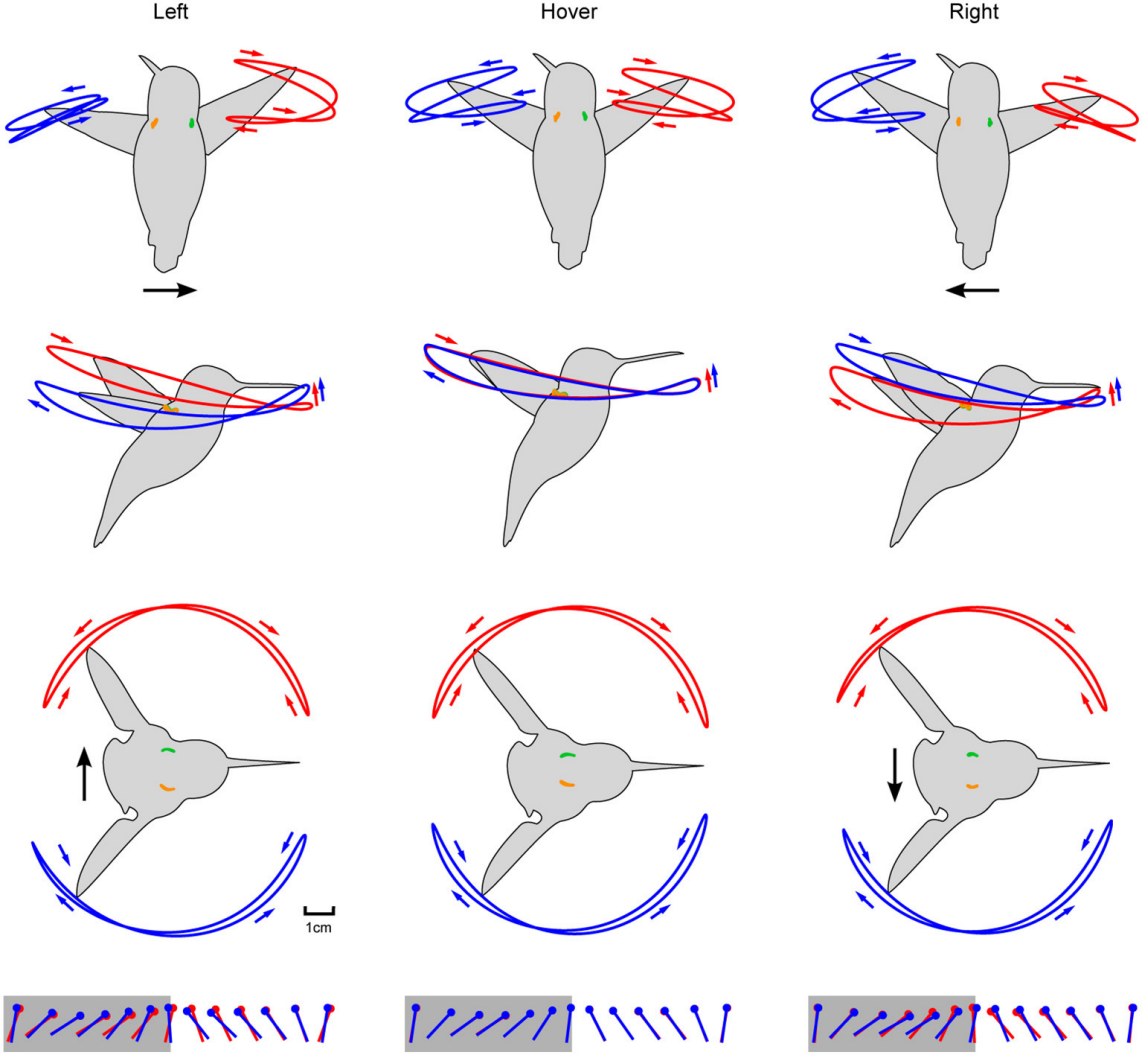
Aligning the wing and body kinematics reveals that hummingbirds are steering against the motion of the laterally moving feeder. Feeder tracking to the left is shown in the left column with rows 1–3 depicting the front, side, and top views, respectively. These same perspectives for hovering and feeder tracking to the right are given in the middle and right columns, respectively. Black arrows represent direction of flight and anatomical features are color coded as in Figure 4B. Stroke direction is indicated by small blue and red arrows. The fourth row depicts the kinematic angle of attack during the downstroke (gray) and upstroke (white).

The incident velocity was calculated as:

(7) $${\vec{V}}_{\text{incident}}={\vec{V}}_{\text{Rgyr}}+{\vec{V}}_{\text{induced}}+{\vec{V}}_{\text{body}}$$

where *V*_body_ is the velocity of the bird (0 for hovering, 0.15 ms^−1^ for lateral flight), and *V*_induced_ is the velocity of the air induced by the motion of the wings. *V*_Rgyr_ is the velocity of the wing at the radius of gyration, calculated from the kinematic data. The radius of gyration is calculated as:

(8) $$R_{\text{gyr}}=r_{2}L$$

where *r*_2_ is the non-dimensional radius of the second moment of the area of the wing (0.499) and *L* is the wing length (0.0525 m). The induced velocity was estimated using the Rankine-Froude model, which assumes a flat stroke plane and a downward directed velocity. Pennycuick (1968) derives two equations for induced velocity, one for hovering and one for forward flight at speeds greater than 1 ms^−1^. Because the speed of our controlled lateral flight trials was much less than 1 ms^−1^ we use the hovering equation for all of our calculations:

(9) $$V_{\text{induced}}=\sqrt{\frac{Mg}{2\rho A_{\text{disk}}}}$$

where *M* is the mass (0.00468 kg), *g* is the gravitational constant, and *A*_disk_ is the area swept out by the actuator disk, estimated by the equation:

(10) $$A_{\text{disk}}=(\frac{1}{180})\Phi_{\text{SP}}\;\pi \;L^{2}$$

where Φ_sp_ is the calculated stroke amplitude, and *L* is the wing length (0.0525 m). To calculate *V*_tip_ and α_aero_ we used the average wingbeat kinematics presented in Figure 5, assuming a 39 Hz wingbeat frequency for hovering and a 41 Hz wingbeat frequency for feeder tracking, as well as a downstroke:upstroke ratio of 48:52 for both hovering and feeder tracking.

The lift acts in the direction of the vector obtained by taking the cross product of the leading edge of the wing and *V*_incident_, in the positive vertical direction, whereas the drag acts in the direction of *V*_incident_. The instantaneous forces were determined for 200 equally spaced time points during the course of the wingbeat, from which the average forces in the global vertical, forward, and lateral directions were calculated.

## Results

### Hummingbirds fly against the direction of feeder motion—wingbeat kinematics

When feeding, hummingbirds inserted their bill into the artificial feeder by as much as 50% of the exposed culmen or as little as a few percent (Figure 4D, Table 1). However, this amount of bill insertion did not differ between hovering and feeder tracking (means: left 24.8%, hovering 19.8%, right 27.7%; *P* = 0.683). Birds tended to face the direction of travel during lateral flight, but we did not analyze this with a mixed model because travel angle (Ψ) has no meaning during hovering flight. The body angle from the lateral perspective (χ_GR, XZ_) was broadly similar across treatments. From the frontal perspective (χ_GR, YZ_), the hummingbirds' bodies were significantly tilted to the right during feeder tracking to left but were close to vertical (90°) during hovering and feeder tracking to the right.

**Table 1:** Mixed model ANOVA (mma) of kinematics: bill percentage inserted in feeder (Bill %), travel angle (Ψ), lateral body angle (χ_GR, XZ_), frontal body angle (χ_GR, YZ_), wing bank angle (*WBA*), and relative wing bank angle (*RWBA*) by maneuver (left lateral flight, hovering, right lateral flight).

		**Mean**				***P*-value**		
	**Left**	**Hovering**	**Right**		**mma**	**L-H**	**R-H**	**L-R**
Bill %	24.8	19.8	27.7		0.683	–	–	–
Ψ (°)	21.9	–	−10.3		–	–	–	–
χ_GR, XZ, DS_ (°)	59.1	66.8	56.5		0.064	–	–	–
χ_GR, XZ, US_ (°)	57.6	65.4	54.5		0.047	0.052	0.015	0.742
χ_GR, YZ, DS_ (°)	3.5	0.2	−1.8		0.009	0.007	0.337	<0.001
χ_GR, YZ, US_ (°)	2.6	0.2	−1.3		0.041	0.068	0.557	0.009
*WBA*_DS_	4.0	0.0	−2.6		<0.001	<0.001	<0.001	<0.001
*WBA*_US_	7.9	−0.6	−7.4		<0.001	<0.001	<0.001	<0.001
*RWBA*_DS_	7.5	0.1	4.4		0.001	<0.001	0.006	0.111
*RWBA*_US_	10.5	−0.4	8.7		<0.001	<0.001	<0.001	0.595

*Bird was included as a random effect within the model. The degrees of freedom for the Bill % ANOVA is 2, 5, and for the other parameters is 2, 8. For models with significant ANOVAs three post-hoc comparisons were made: left vs. hovering (L-H), right vs. hovering (R-H), and left vs. right (L-R). The means are presented along with the P-values for the ANOVA and the three post-hoc comparisons. The P-values for Ψ are not reported because travel angle is not a meaningful measurement during hovering*.

The two wings were highly symmetrical in motion during hovering flight, but were highly asymmetrical during lateral feeder tracking (Figure 4E). Digitized frames from high-speed video allowed us to quantify overall wing motion but not wing deformation. The most salient difference observed during feeder tracking was that the leading wing was elevated relative to the trailing wing during most of the wingbeat (Figure 5) Thus, the wing bank angle (WBA) was tilted opposite to the direction of motion. Moreover, the birds did not maintain perpendicular wing bank angles (RWBA) between wings and the dorsal body axis. These patterns held for both downstroke and upstroke and were observed in almost every wingbeat.

The two wings also differed in the time course of the geometric angle of attack (α) for most of the wingbeat (Figure 5). On average, the change in the angle of attack (wing rotation) was delayed in the leading wing and advanced in the trailing wing during the transition from downstroke to upstroke (supination).

The kinematic patterns and mixed model ANOVA demonstrate that hummingbirds do not orient the stroke plane into the direction of travel during lateral feeder tracking, which suggests that the birds are being dragged by the feeder and orient their aerodynamic force to resist feeder motion.

### Hummingbirds fly against the direction of feeder motion—forces and flow visualization

**Figure 6:**
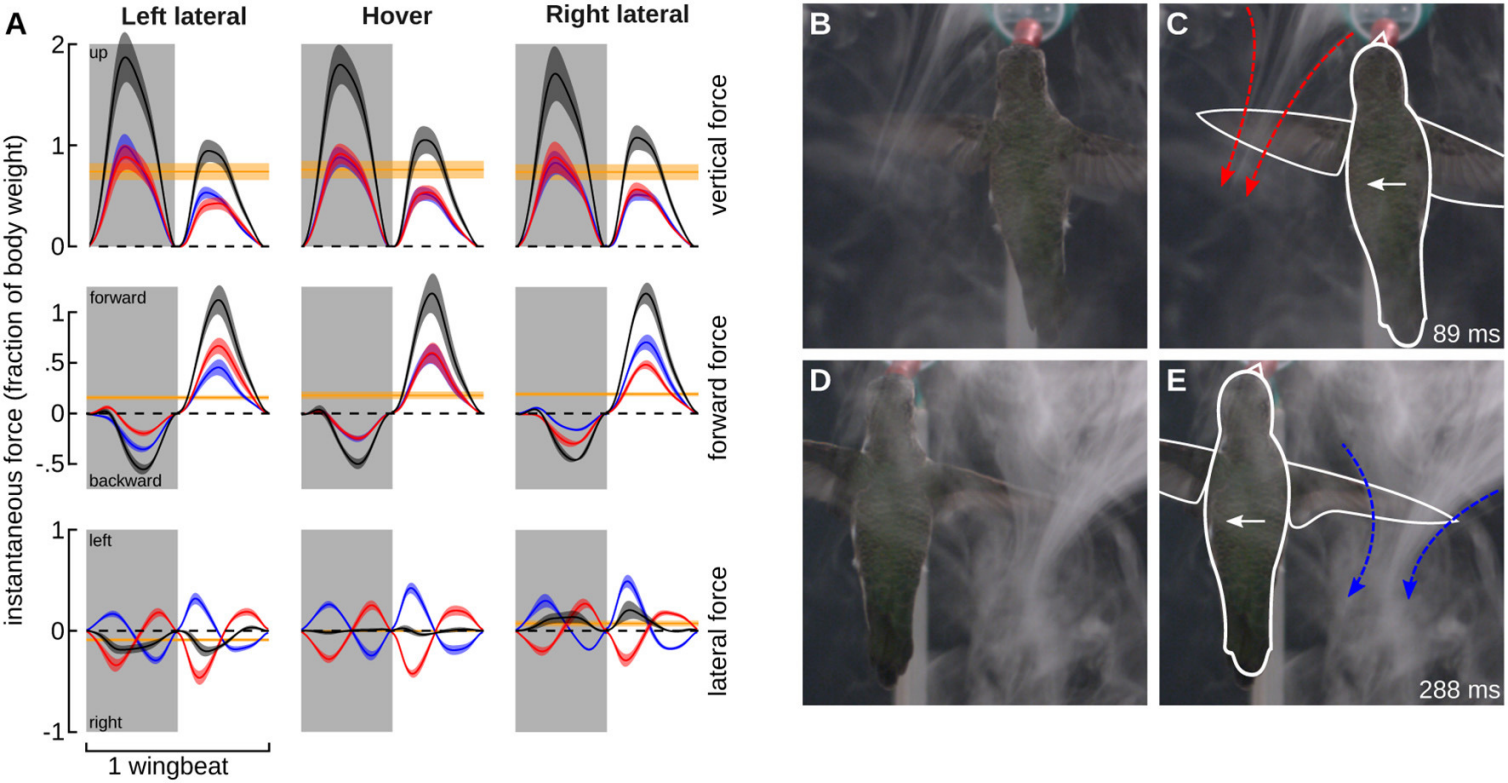
Quasi-steady aerodynamic analysis and smoke visualization demonstrate that hummingbirds “track” a laterally moving feeder by generating flight forces that oppose its motion. **(A)** The instantaneous vertical (top row), forward (middle row), and lateral (lower row) forces were calculated using a blade-element analysis. The forces based on the average wingbeat kinematics (Figure 5) are shown for feeder tracking to the left (left column), hovering (middle column), and feeder tracking to the right (right column). Instantaneous forces generated by the left (red) and right (blue) wings, and the net force (black) are expressed relative to body weight. The net force over the whole wingbeat and along each axes is depicted by the solid yellow line. Lines represent average values and transparent bands indicate the standard errors of the mean calculated across birds. The timing for all average traces is normalized by down- and up-stroke, with each representing 50% of the wingbeat. Downstrokes are represented in gray and upstroke are represented in white. Bird #7 was filmed from the dorsal perspective during feeding tracking to the left in the presence of a CO_2_ plume created by the sublimation of dry ice. The complete video is available in the online Supplementary Materials (Video S2). The frame at 89 ms provides a view of the jet generated by the left (leading) wing immediately after mid-downstroke. This frame is provided twice, once as an unmanipulated image **(B)** and again with the outline of the bird (white) and the jet (red) included **(C)**. The frame at 288 ms from the same video provides a view of the jet generated by the right (trailing, blue) wing immediately after mid-downstroke. Unmanipulated **(D)** and outlined **(E)** images are provided. The feeder was moving at 30 cm/s.

We tested the hypothesis that hummingbirds generate force opposite to the direction of travel during feeder tracking using two methods. The first is a quasi-steady blade-element aerodynamic analysis. The quasi-steady forces in the vertical, thrust, and lateral components are presented for the average wingbeat pattern during feeder tracking to the left, hovering, and feeder tracking to the right, expressed in a bird-centered frame of reference (Figures 6A–C). The quasi-steady analysis for hovering flight largely supports observations from other studies such as left-right symmetry for the vertical and thrust components, asymmetry in force output between the downstroke and upstroke, and 76% weight support (0.76 wb) (Fry et al., 2003; Warrick et al., 2005; Kruyt et al., 2014). There was an unexpected asymmetry between backwards and forwards thrust for all three flight modes. During left lateral flight, the net thrust was 0.16 wb in the forward direction. Net thrust was 0.19 wb in the forward direction during right lateral flight and 0.18 during hovering flight. This result was probably due to wing twist, which was unaccounted for in the kinematic analysis. The proximal section of the wing had a very high angle of attack and the distal section had a much lower angle of attack, but our measure of α was based on a flat plane that incorporated both sections. This likely led to a high estimate for drag and forward thrust during the upstroke.

The left and right wings produce opposite lateral forces, which essentially sum to zero over the course of the full hovering wingbeat. During feeder tracking, the net vertical and thrust components are similar to hovering flight, but the lateral components do not sum to zero, and the net lateral forces are oriented opposite to the direction of travel. During feeder tracking to the left, the average lateral force is 0.09 wb toward the right, and during feeder tracking to the right, the average lateral force is 0.07 wb to the left. These lateral forces closely match mean force estimates from measurements at the feeder in response to visual motion. During visual manipulations with a stationary feeder we measured 0.08 and 0.05 wb to the right and left, respectively. Because the laterally moving birds had non-zero travel angles (21.9° during left lateral flights and 10.3° during right lateral flights), we also calculated the time-averaged quasi-steady force in the direction of travel, which was 0.02 wb to the right during left lateral flight and 0.04 wb to the left during right lateral flight. It is important to note that if the forward thrust component during the upstroke is inflated (see above), then the quasi-steady forces opposite to the direction of travel will be even larger.

We next used flow visualization via sublimation of dry ice to examine the orientation of the jets caused by the wings. This visualization approach works best for the impulse jets caused by the flapping of the wings and is therefore another method to describe the feasibility of quasi-steady mechanisms. Feeder tracking through the smoke revealed the presence of two jets, one under each of the leading and trailing wings (Figure 6B). The primary flows and the resulting momentum impulse were oriented opposite to the direction of travel. Thus, both the quasi-steady analysis and the flow visualization support the hypothesis that the birds are being dragged by the feeder during lateral feeder tracking, despite vastly different levels of bill insertion during different trials (Figure 4D).

## Discussion

Docking at the flower is an important behavior for hummingbirds, whose specialization for hovering is required for holding station when feeding on floral nectar (Altshuler and Dudley, 2002). Maintaining docked position is complicated by dynamic and unpredictable natural environments, in which target flowers and background foliage move with the wind. To study how hummingbirds use visual and tactile feedback to hold body position in dynamic situations, we measured responses of docked hummingbirds to two experimental scenarios: (1) a stationary feeder and moving visual background and (2) a laterally moving feeder and a blank visual background. We showed that docking involves physical interactions with the feeder (Figure 2), and that docked hummingbirds attempt to move their head or body to match the direction and speed of background visual motion (Figure 3), even though they are in contact with a stationary feeder. When the feeder moves, but the visual environment remains stationary, the hummingbirds do not fly laterally to track the feeder but instead produce aerodynamic force in opposition to the feeder's motion (Figures 5, 6). These results suggest that docked hummingbirds primarily use visual feedback from the background to maintain stationary body orientation and position during feeding, and attempt to do so even when the feeder is moving.

We found little evidence that tactile information available during docked hovering significantly altered the hummingbird response to background visual motion. Fusion of tactile and visual information, or a shift from background (visual) to feeder based (visual and tactile) cues, was not evident during stationary feeding with a horizontally moving background and also not during experiments where the visual background was blank but the feeder moved laterally. Tactile information can improve stability at very low levels of force (Marsden et al., 1981; Holden et al., 1994; Jeka and Lackner, 1994), and we showed docking involved contact between the hummingbird's bill and the feeder. We therefore had predicted that the contact with the feeder would be sufficient to allow hummingbirds to ignore the visual cues from the background.

In our experiments, hummingbirds were not able to use visual or tactile cues from the feeder to ignore the visual background. However, our measures of hummingbird response were not sensitive enough to completely rule out small effects of tactile information, and we cannot compare the force measurements and estimates in this study with behavioral responses of undocked hummingbirds that do not have access to tactile information from a feeder (Goller and Altshuler, 2014). We therefore conclude that background visual cues are the dominant source of information for docked position control. Visual and tactile cues from the feeder could have a small influence on docked hovering and could be more important at other motion speeds or in other motion directions. For instance, the visual motion and feeder motion speeds we tested may have created a sensory context where tactile information was too unreliable, causing the hummingbirds to primarily rely on visual cues. Future experiments examining slower speeds of visual motion or feeder translation might determine whether tactile information plays a larger role in those contexts. The absence of a significant push response to a visual pattern moving at 2°/s for example may indicate the contribution of stabilizing tactile information at slow speeds.

The result that hummingbirds are not tracking the feeder is surprising and novel. Our results for docked hovering show that hummingbirds use the visual background to adjust body position, just as was previously shown for undocked hovering where no flower interaction is involved (Goller and Altshuler, 2014; Ros and Biewener, 2016). This is not the case for hawkmoths, which similarly use visual motion to stabilize body position, but also use visual cues from feeders to track oscillations up to 5 Hz in forward-backward directions (Farina et al., 1994) and 2–3 Hz laterally (Sponberg et al., 2015; Stöckl et al., 2017). Hummingbirds dock with a rigid bill instead of a flexible proboscis, and perhaps this structural difference enables hummingbirds to resist flower motion, whereas the hawkmoths have to track to remain docked. Hummingbirds also have greater body masses than the comparable moths (Henningsson and Bomphrey, 2013), which may enable them to effectively oppose flower motions. The smallest hummingbirds (~2 g) are similar size to the large *Manduca sexta* moths, and it would be interesting to study whether these hummingbirds also track flower motions like the moths, or hold a stationary position like the Anna's hummingbird.

Our results also show that studies using rigid feeders to elicit flight maneuvers in hummingbirds must carefully assess what flight behavior hummingbirds are using to maintain contact with the feeder, and what the contribution of the feeder to the flight maneuver may be (Altshuler et al., 2012; Sapir and Dudley, 2012; Ravi et al., 2015; Read et al., 2016). Hummingbirds are able to perform the lateral flight maneuvers needed to track a laterally moving feeder, as shown by the shuttle displays of black-chinned hummingbirds (Feo and Clark, 2010). It is unclear why they would not fly laterally to match the feeder. One possible explanation is that in the laboratory, the direction reversals of the motorized feeder are difficult to track, so hummingbirds have learned an alternative strategy to simplify the task. Future experiments examining the differences between sensory integration for continuous and dynamic motion stimuli could provide important insight into behavior control. Regardless, our finding that hummingbirds generate force opposite to prediction is yet another example of how tethering animals to study flight kinematics can alter the flight behavior the technique was originally designed to measure (Kutsch and Stevenson, 1981; Fry et al., 2005).

We used the physical connection between the flower and the docked hummingbird's bill to quantify the strength of a hummingbird's response to moving visual backgrounds. We presented two patterns—dot-fields and gratings—and moved them at a range of speeds, then compared the loads imparted on the feeder by the hummingbird. Previous work showed that undocked hummingbirds drift to stabilize motion of black-and-white gratings in all directions but the speed tuning of these responses was not measured (Goller and Altshuler, 2014). A recent study on a key avian visual motion processing nucleus, the lentiformes mesencephali (LM), found uniform responses to different directions of dot-field motion in hummingbirds, which is unlike other birds studied to date (Gaede et al., 2017). For directional loading at a single speed, we measured a significant difference between the response to laterally moving gratings and dot-fields at 12°/s. The difference in response to the two patterns suggests that properties of the patterns besides motion direction and speed may be important for hummingbird behavior. For example, visual expansion of the vertical bars could be an important cue, as is described for forward flight (Dakin et al., 2016). Alternatively, the low spatial frequency of the grating we used could be attenuating the motion signal in comparison with a dot-field, so further tests with higher spatial frequencies could prove informative. Gaede et al. also showed that hummingbird LM neurons were tuned to higher speeds of visual motion than pigeon and zebra finch LM neurons. Our behavioral results for both dot-field and grating motion suggest that hummingbirds do not respond strongly to slow motion (less than 12°/s) and the behavioral response range from 12 to 84°/s matches well the sensitivity of LM neurons. Does LM neuron speed tuning explain the absence of a slow-speed response or is tactile information important for position control in those cases?

Sensory integration of visual and tactile signals warrants future exploration because we currently cannot explain how slow motions are processed, even though slow visual motion speed was predicted to be especially important for hovering (Iwaniuk and Wylie, 2007). How sensory information is used to control flight responses to slow feeder and visual background motion (<12°/s) remains to be investigated. Our results suggest that there is little influence of physical or tactile information on either feeder tracking or docked hovering position control at moderate to high speeds of visual motion and feeder translation. Instead, hummingbirds use visual information to maintain their body position and orientation, either by pushing against a stationary feeder in response to visual motion, or producing aerodynamic force to resist or counter the force applied by a laterally moving feeder. In contrast to hovering moths that track feeder motion, hummingbirds apply counter-forces or torques that may hold flowers in place, suggesting that moths and hummingbirds may have two different sensory strategies to accomplish a similar foraging behavior. In turn, the different strategies during docked flight may also have implications for flower corolla morphology, which must withstand repeated visits from their pollinators.

## Ethics statement

This study was carried out in accordance with the recommendations of US federal regulations and the Guide for the Care and Use of Laboratory Animals, as well as the Canadian Council on Animal Care. The protocol was approved by the Institutional Animal Care and Use Committee of the California Institute of Technology and the Animal Care and Use Committee at the University of British Columbia.

## Author contributions

BG, MD, and DA conceived and designed the experiments. BG and DA collected the data. BG, PS, KM, and DA analyzed the data and wrote the manuscript. All authors edited the manuscript.

### Conflict of interest statement

The authors declare that the research was conducted in the absence of any commercial or financial relationships that could be construed as a potential conflict of interest.

## Supplementary material

Video S1 A movie of a bird performing controlled lateral flight to the left while feeding from a moving feeder. The feeder was moving at 0.15 m/s and the video is slowed down 40x.

Video S2 A hummingbird feeding from a moving feeder at 30 cm/second through a plume of CO_2_. The inert plume was created by sublimation of dry ice. The video is slowed down 40x.
